# From Species to Varieties: How Modern Sequencing Technologies Are Shaping Medicinal Plant Identification

**DOI:** 10.3390/genes16010016

**Published:** 2024-12-26

**Authors:** Mingcheng Wang, Haifeng Lin, Hongqiang Lin, Panyue Du, Shuqiao Zhang

**Affiliations:** 1 Institute for Advanced Study, Chengdu University, No. 2025 Chengluo Road, Chengdu 610106, China; 2 Engineering Research Center of Sichuan-Tibet Traditional Medicinal Plant, Chengdu 610106, China; 3 School of Food and Biological Engineering, Chengdu University, Chengdu 610106, China; 13629003176@163.com (H.L.); 17781112743@163.com (P.D.); zhangshuqiao196@163.com (S.Z.); 4 Sichuan Wolong National Natural Reserve Administration Bureau, Wenchuan 623006, China; 13348986271@163.com

**Keywords:** medicinal plant identification, advanced sequencing technologies, DNA barcoding and super-barcoding, multi-omics integration, innovative genomic solutions

## Abstract

Background/Objectives: Modern sequencing technologies have transformed the identification of medicinal plant species and varieties, overcoming the limitations of traditional morphological and chemical approaches. This review explores the key DNA-based techniques, including molecular markers, DNA barcoding, and high-throughput sequencing, and their contributions to enhancing the accuracy and reliability of plant identification. Additionally, the integration of multi-omics approaches is examined to provide a comprehensive understanding of medicinal plant identity. Methods: The literature search for this review was conducted across databases such as Google Scholar, Web of Science, and PubMed, using keywords related to plant taxonomy, genomics, and biotechnology. Inclusion criteria focused on peer-reviewed studies closely related to plant identification methods and techniques that contribute significantly to the field. Results: The review highlights that while sequencing technologies offer substantial improvements, challenges such as high costs, technical expertise, and the lack of standardized protocols remain barriers to widespread adoption. Potential solutions, including AI-driven data analysis and portable sequencers, are discussed. Conclusions: This review provides a comprehensive overview of molecular techniques, their transformative impact, and future perspectives for more accurate and efficient medicinal plant identification.

## 1. Introduction

The accurate identification of medicinal plants is fundamental to ensuring the quality, efficacy, and safety of herbal medicines [1]. While medicinal plants have been used for centuries in traditional medicine systems, their therapeutic benefits are now widely recognized in modern pharmacology [2,3,4]. However, the consistency of these benefits relies on the precise identification and authentication of plant materials. Misidentification can lead to ineffective treatments or, in severe cases, adverse health effects from toxic compounds [5]. The risks of adulteration and substitution have increased with globalization and the growing demand for herbal products, emphasizing the need for more robust identification techniques. Traditional identification methods, such as morphological analysis, anatomical analysis, and chemical fingerprinting, cannot attain the precision required in current quality control standards.

Morphological and anatomical traits have long been the foundation of plant identification [6,7]. However, these methods face significant limitations, especially when distinguishing closely related species or varieties. Factors such as environmental conditions, developmental stages, or phenotypic plasticity can influence traits like leaf shape and tissue structure, leading to misidentifications [8,9,10]. Similarly, chemical fingerprinting, which analyzes the chemical composition of plant extracts, is influenced by variables like harvest time, geographic location, and cultivation practices [11,12]. These traditional methods are time-consuming and require specialized expertise, which is not always available. Consequently, these methods often fail to consistently distinguish between morphologically similar but genetically distinct species or between varieties with subtle phenotypic differences that impact medicinal quality. These challenges underscore the urgent need for more reliable, precise, and efficient identification techniques.

Modern sequencing technologies have emerged as powerful tools for identifying and authenticating medicinal plants at both the species and variety levels, overcoming the challenges of traditional methods. Molecular markers, DNA barcoding, and high-throughput sequencing technologies have revolutionized the field by providing molecular-level resolution unattainable through traditional means [13,14,15]. By analyzing specific DNA regions or entire genomes, these techniques offer high accuracy in species differentiation, detecting genetic diversity, and identifying plant varieties. Next-generation sequencing (NGS) and third-generation sequencing (TGS) platforms ensure rapid, large-scale analysis of genetic materials [16,17], facilitating the accurate identification of complex samples or mixed herbal products. These advancements have significant implications for quality control, biodiversity conservation, and the sustainable management of medicinal plant resources.

Despite their advantages, modern sequencing technologies also present several challenges. High-throughput sequencing generates vast volumes of data, necessitating sophisticated bioinformatics tools for analysis, storage, and interpretation [18]. The implementation cost of these technologies, coupled with the expertise needed for their operation, can be prohibitive for smaller laboratories, particularly in regions where medicinal plants are widely used. Furthermore, a lack of standardized protocols and reference databases remains a major hurdle, impacting the reliability and reproducibility of results across different laboratories [19]. Overcoming these obstacles will require continued research, collaboration, and investment to fully integrate sequencing technologies into routine medicinal plant identification practices.

This review focuses on the transformative role of modern sequencing technologies in advancing the identification of medicinal plant species and varieties. It aims to examine the current techniques, evaluate their advantages and limitations, and identify opportunities for future integration into herbal medicine quality control. Specifically, the study aims to (1) highlight the potential of molecular methods to address challenges in species and variety identification, (2) explore the integration of sequencing technologies into practical applications, and (3) outline key areas for further research to enhance accuracy and efficiency in plant identification.

## 2. Materials and Methods

The literature search was conducted using Google Scholar, Web of Science, and PubMed. Keywords included “medicinal plant identification”; “DNA barcoding”; “multi-omics approaches”; and related terms in plant taxonomy, genomics, and biotechnology. While Preferred Reporting Items for Systematic Reviews and Meta-Analyses (PRISMA) guidelines were not followed, the literature was selected based on relevance, quality, and publication date. The inclusion criteria were (1) studies published in peer-reviewed journals; (2) research closely related to the review’s topic; and (3) studies that contribute significantly to plant identification methods and techniques.

## 3. Historical Approaches to Medicinal Plant Identification

**Figure 1:**
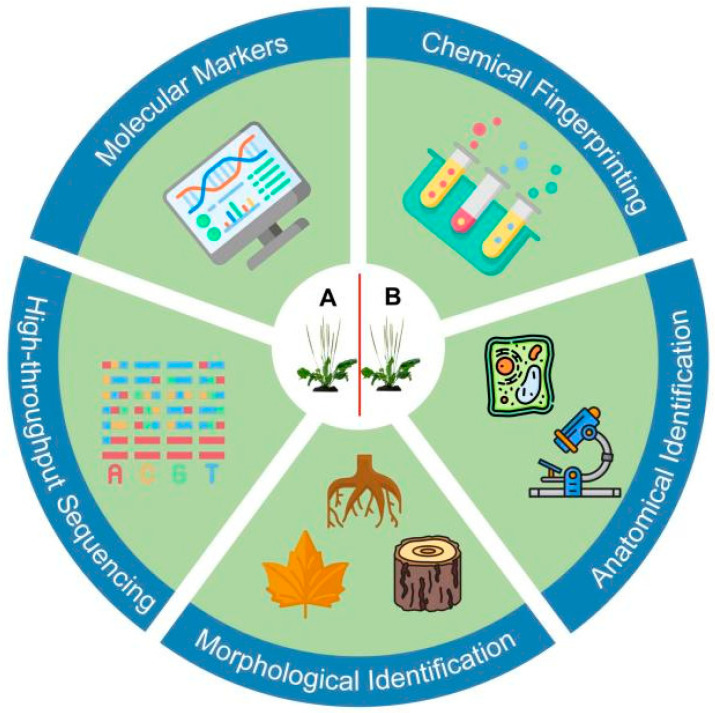
Overview of traditional and modern techniques for medicinal plant identification.

Traditional methods such as morphological analysis, anatomical studies, and chemical fingerprinting have played key roles in identifying and differentiating plant species and varieties (Figure 1). However, they also present limitations in terms of reliability and precision.

### 3.1. Morphological and Anatomical Identification

For centuries, botanists and herbalists have relied on morphological traits—such as leaf shape, flower structure, root type, and seed morphology—to distinguish medicinal plants. While often effective, this approach faces significant challenges, particularly with closely related species. Environmental factors—such as soil type, climate, and altitude—can induce phenotypic plasticity, where plants alter their morphology and physiology in response to environmental conditions [8,20]. For instance, *Taraxacum officinale* F.H.wigg. exhibits changes in photosynthetic performance, flowering time, and biomass in response to water availability [21]. Developmental differences also influence plant traits, leading to variability at different life stages [9]. These factors make morphology-based identification unreliable for species or varieties with overlapping traits influenced by environmental conditions.

Anatomical analysis, which examines internal structures like vascular tissues and cell types, can provide supplementary information to support morphological identification [7]. However, anatomical traits are also susceptible to environmental variability [10]. For example, under drought stress, *Thymus* × *citriodorus* (Pers.) Schreb. shows increased root development and reduced shoot growth, along with stomatal closure to minimize water loss [22]. Additionally, anatomical methods are time-consuming and require specialized expertise, limiting their widespread application.

Although valuable, the reliance on environmental conditions and specialized skills makes morphological and anatomical approaches less reliable, especially for distinguishing closely related species.

### 3.2. Chemical Fingerprinting

Chemical fingerprinting techniques emerged in the late 20th century to address the limitations of morphological and anatomical approaches [23]. These techniques analyze the chemical composition of plant extracts using methods such as high-performance liquid chromatography (HPLC) [24], gas chromatography–mass spectrometry (GC-MS) [25], and thin-layer chromatography (TLC) [26]. These methods detect bioactive compounds unique to specific species and varieties, providing a more precise basis for plant identification. For example, GC-MS combined with chemometric tools can differentiate psychoactive Solanaceae plants like *Datura metel* L., *Scopolia lurida* Dunal, and *Atropa belladonna* L. based on their atropine and scopolamine concentrations, which is particularly useful in forensic analysis [27]. Additionally, HPLC fingerprint analysis can differentiate *Coptidis* species and their geographical origins, ensuring proper use in traditional Chinese medicine [28].

However, chemical fingerprinting also has several challenges. The chemical composition of a plant can be influenced by factors like harvest time, geographic location, and cultivation practices [11,12]. For instance, *Coptis chinensis* Franch. shows significant variability in alkaloid content depending on the plant organ and growing environment [29]. Similarly, *Salvia miltiorrhiza* Bunge exhibits variation in chemical constituents across different growth stages and plant parts, complicating its identification [30]. Closely related species can also produce similar sets of compounds, such as the overlapping chemical profiles of *Cinnamomum verum* J.Presl and *Cinnamomum cassia* (L.) J.Presl [31], making distinctions based solely on chemical analysis challenging. Moreover, chemical fingerprinting is labor-intensive and requires sophisticated instrumentation, limiting its practicality for routine use.

### 3.3. Limitations of Traditional Methods

Traditional methods for medicinal plant identification are fraught with challenges, particularly for closely related species or varieties. Morphological and anatomical methods are subjective, relying heavily on the skill and experience of the identifier, which can lead to inconsistent results. Chemical fingerprinting, while more objective, is influenced by external factors affecting chemical markers and requires highly controlled conditions for reproducibility. The identification of plants like *Echinacea* or *Panax* species, which often have overlapping traits, can result in misidentification, potentially compromising therapeutic efficacy or posing risks to consumer safety [32,33]. Given the growing demand for herbal products and increased incidents of adulteration, the need for more precise identification techniques is greater than ever [5].

### 3.4. Transition to Molecular Methods

There has been a shift toward molecular techniques for plant identification to overcome the limitations of traditional approaches. Molecular methods, such as DNA barcoding and NGS, offer a level of precision and reproducibility not attainable by traditional methods. By analyzing specific genetic sequences, these methods accurately identify species regardless of environmental conditions or phenotypic variability. This transition represents a significant advancement, paving the way for more reliable quality control in medicinal plant research.

The adoption of molecular techniques has not only improved identification accuracy but also facilitated the discovery of novel species and the conservation of endangered taxa [34,35]. With their increasing accessibility, integrating these technologies into routine quality control systems for herbal medicines is expected to enhance standardization, safety, and efficacy [36]. The shift from traditional to molecular methods reflects the evolving demands of the field, emphasizing the need for precision, reproducibility, and efficiency in medicinal plant identification.

## 4. Advancements in DNA-Based Identification Methods

**Figure 2:**
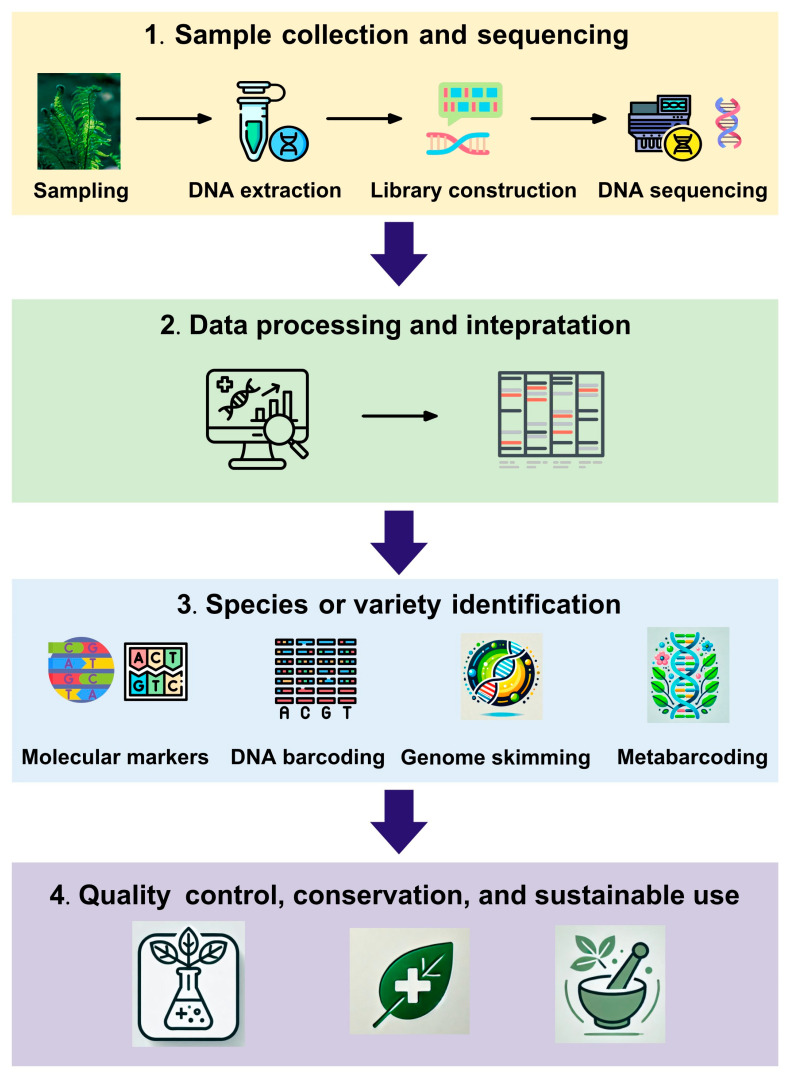
Commonly used DNA-based identification pipeline for medicinal plants.

As traditional identification methods face growing limitations, DNA-based techniques have emerged as powerful tools for the accurate and efficient identification of medicinal plant species and varieties (Figure 2). This section explores the use of molecular markers, DNA barcoding, and super-barcoding, emphasizing their strengths, weaknesses, and recent developments.

### 4.1. Molecular Markers for Species and Variety Identification

Molecular markers, including simple sequence repeats (SSRs), single-nucleotide polymorphisms (SNPs), random amplified polymorphic DNA (RAPD), and amplified fragment length polymorphism (AFLP), provide an objective and reliable approach to plant identification [13]. These markers utilize variations in DNA sequences to enable precise genetic differentiation [37]. For example, SSR markers were used to analyze 800 lines of *Capsicum* collected from Central and South America, resulting in the grouping of 192 lines into five clusters based on polymorphism [38]. Similarly, RAPD markers effectively differentiated two varieties of *Silybum marianum* (L.) Gaertn. that are difficult to distinguish in dried conditions through unique banding patterns [39].

Each molecular marker has its advantages and challenges. SSR markers are highly polymorphic and abundant, making them ideal for distinguishing closely related species and varieties. However, developing SSR markers is labor-intensive and requires prior genomic information [40]. SNP markers, based on single nucleotide changes, exhibit high frequency and stability, but their usage demands significant expertise and resources, limiting accessibility for smaller laboratories [41]. RAPD allows polymorphism detection without prior genomic knowledge but lacks reproducibility [42]. Meanwhile, AFLP generates high levels of polymorphism but requires significant optimization, which limits its widespread adoption [43]. As such, while molecular markers are useful for genetic differentiation, their application is still limited by challenges regarding reproducibility, resource requirements, and expertise.

### 4.2. DNA Barcoding and Super-Barcoding: Principles and Applications

DNA barcoding has gained popularity due to its simplicity and efficiency in species identification using short, standardized DNA sequences [14]. DNA barcoding enables effective species identification by sequencing a specific gene region that is highly conserved within species yet variable between species. Commonly used barcode regions for plants include *rbcL*, *matK*, and the Internal Transcribed Spacer (ITS). The *rbcL* gene, encoding the subunit of ribulose-1,5-bisphosphate carboxylase, is frequently used due to its universality and ease of amplification, although its limited variability can hinder the differentiation of closely related species [44]. The *matK* gene provides higher discriminatory power but is more difficult to amplify. The ITS region is highly variable and located between ribosomal RNA genes, making it effective for distinguishing closely related species and thus often preferred in barcoding studies [45].

Super-barcoding is an evolution of traditional DNA barcoding, which utilizes longer DNA sequences or entire genomic regions to enhance resolution [46]. Super-barcoding enables the differentiation of species that are indistinguishable through traditional barcoding by leveraging NGS technologies, particularly in complex herbal mixtures containing multiple species. Unlike conventional barcoding, which targets short loci, super-barcoding employs whole chloroplast genomes or large nuclear genomic regions, providing enhanced accuracy and discriminatory power. This approach is particularly advantageous for resolving taxonomically challenging groups, identifying cryptic species, and analyzing samples with high genetic diversity. For instance, super-barcoding has been used in tea plants (*Camellia* sect. *Thea*) to successfully distinguish morphologically similar species previously difficult to identify using traditional barcoding [47]. Similarly, a study on *Fritillaria* species demonstrated that using whole chloroplast genomes as super-barcodes could more effectively distinguish closely related medicinal species than traditional barcoding methods [48]. The use of super-barcoding in plant species identification enhances taxonomic precision but aids biodiversity assessments and conservation efforts through comprehensive genetic analysis.

### 4.3. Advantages of DNA Barcoding over Traditional Methods

DNA barcoding offers numerous advantages over traditional identification methods. Unlike morphological or chemical approaches, which can be influenced by environmental conditions, DNA barcoding relies on stable genetic sequences, ensuring consistent results across different samples and conditions. This stability is particularly beneficial in the herbal medicine industry, where accurate identification is essential for quality control. Additionally, DNA barcoding is cost-effective and efficient, particularly with advancements in high-throughput sequencing technologies, which enable simultaneous analysis of multiple samples, significantly reducing labor and material costs. The development of comprehensive reference databases like the Barcode of Life Data System (BOLD) [49] has facilitated the adoption of DNA barcoding by providing accessible reference sequences for thousands of species, enhancing the efficiency of species identification.

### 4.4. Challenges and Limitations of DNA Barcoding and Super-Barcoding

Despite their advantages, DNA barcoding and super-barcoding methods have certain limitations. While DNA barcoding is effective at the species level, it often lacks the resolution to differentiate closely related varieties within the same species [50]. For example, standard barcode regions may not distinguish between different therapeutic varieties of *Panax ginseng* C.A.Mey [51]. Furthermore, the reliability of DNA barcoding depends heavily on the availability of high-quality reference databases. Incomplete or poorly curated databases can lead to misidentification, especially for closely related species [52]. Although super-barcoding offers enhanced resolution, it requires sophisticated bioinformatics tools and expertise, which may not be available to all laboratories, particularly in regions where medicinal plants are widely used. This limits its accessibility, especially to smaller facilities.

In conclusion, DNA-based identification methods, such as molecular markers, DNA barcoding, and super-barcoding, have greatly improved the reliability and efficiency of medicinal plant identification. However, challenges such as distinguishing closely related varieties and limitations in data analysis remain key areas for future development. Advancing technology and expanding comprehensive reference databases will be crucial for maximizing the potential of these DNA-based approaches.

## 5. NGS in Plant Identification

With the limitations of traditional identification methods, NGS technologies have become transformative tools for plant identification. Unlike molecular markers and DNA barcoding that target specific loci, NGS allows for comprehensive sequencing of large genomic regions, providing unmatched accuracy and scalability [16]. Platforms like Illumina and Ion Torrent enable the rapid, simultaneous sequencing of millions of DNA fragments, significantly enhancing the analysis of complex herbal mixtures and the authentication of medicinal plant species (Table 1). NGS technologies are well known for their scalability, precision, and cost-effectiveness, enabling detailed genetic analysis even for complex and mixed samples.

### 5.1. Overview of NGS Technologies

NGS technologies, such as Illumina and Ion Torrent, have revolutionized plant identification by enabling high-throughput sequencing at relatively low costs. Illumina sequencing uses reversible dye terminators to simultaneously process millions of fragments, making it suitable for large-scale projects like whole-genome sequencing. In contrast, the Ion Torrent platform detects hydrogen ions released during sequencing, providing rapid results suitable for projects needing quick turnaround. These platforms generate detailed genetic profiles, essential for accurately identifying plant species and varieties, especially in complex herbal products [53]. Unlike molecular markers and traditional DNA barcoding, NGS sequences entire genomic regions, detecting novel markers and distinguishing species that traditional methods cannot. This comprehensive sequencing capacity provides deeper insights, significantly improving the resolution of plant identification.

### 5.2. Genome Skimming for Plant Identification

Genome skimming is an NGS technique that targets repetitive, abundant genomic regions, such as plastid genomes, mitochondrial DNA, and high-copy nuclear DNA like ribosomal RNA genes [15,54]. Unlike DNA barcoding, which targets short loci, genome skimming generates low-coverage data for entire plastid genomes, enabling high-resolution species identification, even among closely related taxa. This method is cost-effective, rapid, and ideal for studies needing reliable phylogenetic information or species identification without extensive sequencing. Genome skimming has been widely applied in plant systematics, biodiversity studies, and herbal quality control to identify multiple species within complex mixtures and ensure product authenticity. For instance, Liu et al. [55] used genome skimming to develop genomic resources for the genus *Celtis* (Cannabaceae), uncovering phylogenetic relationships and providing valuable data for conservation biology. Similarly, Zhou et al. [56] used genome skimming to accurately differentiate *Paris yunnanensis* Franch. from its congeneric species *Paris liiana* Y.H.Ji, even in processed materials, thereby ensuring authenticity and quality control of commercial products.

The primary advantage of genome skimming is its ability to quickly generate genomic data with minimal sequencing depth, reducing both time and cost. By targeting highly repetitive regions, genome skimming provides sufficient information for species distinction, making it effective for quality control in herbal products and species identification in mixed samples. This approach extends the concept of super-barcoding by providing greater data depth, facilitating the identification of species that traditional barcoding cannot distinguish.

### 5.3. Metabarcoding for Complex Herbal Mixtures

Metabarcoding is another notable NGS application that enables simultaneous amplification and sequencing of multiple barcode regions to identify numerous species in complex herbal mixtures [57]. Unlike traditional barcoding, which targets a single locus, metabarcoding employs a combination of markers like ITS and *matK*, enabling the detection of multiple species in mixed samples [58]. A recent review highlighted metabarcoding’s ability to amplify multiple DNA barcodes using universal PCR primers, which facilitates the efficient identification of species in complex samples [59]. This approach is particularly useful for authenticating herbal products, detecting contaminants, and preventing adulteration—capabilities beyond standard barcoding methods. Metabarcoding allows for rapid and efficient analysis of complex samples, supporting regulatory compliance and maintaining product quality. For example, Urumarudappa et al. [60] used DNA metabarcoding to assess the species composition of 39 Thai herbal products on the National List of Essential Medicines. Their study highlighted the effectiveness of DNA metabarcoding in identifying undeclared plant species, ensuring the authenticity of highly processed multi-ingredient herbal products. In another study, Seethapathy et al. [61] used DNA metabarcoding to analyze 79 Ayurvedic herbal products on the European market, uncovering widespread issues with product quality, including species substitution and undeclared fillers. This underscores the value of DNA metabarcoding in quality control for complex herbal products.

### 5.4. Benefits and Challenges of NGS in Plant Identification

The scalability of NGS makes it ideal for large-scale species identification projects and the development of comprehensive reference databases like GenBank, the European Nucleotide Archive (ENA), the DNA Data Bank of Japan (DDBJ), and the Global Biodiversity Information Facility (GBIF). These databases are essential for ensuring the authenticity of medicinal plant materials, particularly in biodiversity-rich regions where many species remain undocumented. NGS’s ability to detect low-abundance species in complex mixtures surpasses the capabilities of traditional molecular markers and DNA barcoding, thus contributing significantly to the conservation and sustainable use of medicinal plant resources [1].

Despite its numerous advantages, the adoption of NGS technologies faces several challenges. While sequencing costs have significantly decreased, the initial investment in sequencing platforms and computational infrastructure remains high. Moreover, the vast volume of data generated requires advanced bioinformatics tools and expertise, which can be limiting for smaller laboratories, especially in developing regions. The quality of results also relies heavily on the availability of comprehensive reference databases, which are still lacking for many medicinal plant species.

In summary, NGS technologies, including genome skimming and metabarcoding, have significantly advanced medicinal plant identification by providing scalable, accurate, and efficient tools for analyzing complex samples. While molecular markers and traditional barcoding have paved the way, NGS offers a more in-depth solution, overcoming many of the limitations of earlier methods. Addressing challenges such as high costs, bioinformatics complexity, and the need for standardized databases will be crucial for the broader adoption of these technologies in routine plant identification and quality control processes.

## 6. TGS in Plant Identification

TGS technologies, including platforms like Pacific Biosciences (PacBio) and Oxford Nanopore Technologies, have transformed plant genomics by enabling the analysis of complex and repetitive genomic regions that are challenging to resolve with short-read sequencing [17]. This section discusses the advantages of TGS, its applications in plant genomics, and its potential for medicinal plant identification.

### 6.1. Introduction to TGS Platforms

TGS technologies were developed to address the limitations of NGS, particularly its short read lengths [62]. Platforms such as PacBio’s Single-Molecule Real-Time (SMRT) sequencing and Oxford Nanopore sequencing produce reads exceeding 100 kilobases, which are essential for accurately assembling complex genomes, identifying structural variations, and detecting repetitive regions common in medicinal plant genomes. PacBio SMRT sequencing generates continuous, uninterrupted long reads, while Oxford Nanopore sequencing uses electrical signals as DNA passes through nanopores, ensuring real-time sequencing while offering portability, making it ideal for field-based plant identification.

### 6.2. Advantages of TGS for Plant Genomics

A major advantage of TGS is its ability to address genome complexity. Many medicinal plants, such as *Lilium davidii* var. *unicolor* (Hoog) Cotton, *Allium fistulosum* L. and *Taxus wallichiana* Zucc., possess large, highly repetitive genomes that are not ideal for short-read sequencing. The recent chromosome-level assembly of *Lilium davidii* var. *unicolor*, one of the largest plant genomes sequenced to date, demonstrated that TGS technologies like Nanopore can effectively overcome these challenges by generating long reads suitable for the assembly of large, complex genomes [63]. TGS provides superior coverage of repetitive regions, resulting in more accurate genome assemblies and reducing misassemblies. This is particularly beneficial for polyploid plants with multiple gene copies that complicate sequencing. For example, the hexaploid genome of *Hibiscus syriacus* L. was successfully assembled using PacBio and Oxford Nanopore platforms, demonstrating the capability of TGS to handle complex polyploid genomes [64].

TGS is also highly effective in detecting structural variations (SVs)—large-scale genomic changes like insertions, deletions, and translocations—that are critical for understanding the genetic basis of medicinal properties in plants. SVs can influence secondary metabolite biosynthesis, affecting the therapeutic value of medicinal plants [65]. Short-read sequencing often misses these variations due to limited read lengths, but long reads can span entire SVs, providing a more complete genomic picture. For instance, the chromosome-scale genome assembly of *Artemisia argyi* H.Lév. & Vaniot using PacBio sequencing revealed complex SVs, including those resulting from recent lineage-specific whole-genome duplications, offering detailed insights into subgenome evolution and terpenoid biosynthesis pathways [66].

Moreover, TGS can reveal subtle genetic differences at the variety level, which is useful for distinguishing medicinal plant varieties that may have similar sequences in standard barcode regions but differ in non-coding or regulatory regions. These differences significantly impact the production of bioactive compounds and medicinal quality. For example, different varieties of medicinal *Cannabis sativa* L. may have similar barcode sequences but exhibit significant variations in regulatory regions that alter their chemical profiles [67].

### 6.3. Applications in Identifying Complex or Hybrid Genomes

TGS effectively characterizes complex or hybrid genomes, common in medicinal plants due to hybridization and polyploidy. Hybrid species, such as those in the genus *Salvia*, often possess genomic regions inherited from distinct parental species, making their identification challenging with traditional methods [68]. Long reads from TGS can effectively differentiate these genomic regions, enabling comprehensive genetic profiling and precise hybrid species identification. Furthermore, TGS is also well suited for detecting heterozygosity and gene duplications, which are common in medicinal plants [69]. These genetic features can influence the synthesis of secondary metabolites crucial plant’s medicinal efficacy [70]. By offering a deeper understanding of genetic structures, TGS supports the selection and breeding of medicinal plant varieties with desirable traits, enhancing the production of high-quality herbal products [71].

### 6.4. Role of TGS in Enhancing Medicinal Plant Authentication

TGS holds great potential for advancing the identification and authentication of medicinal plants. By resolving complex and repetitive genomic regions, TGS enhances the precision of species and variety identification [72]. Its ability to detect SVs and subtle regulatory differences makes it an invaluable tool for distinguishing medicinal plant varieties that, despite sharing similar genetic backgrounds, exhibit notable phenotypic and pharmacological differences. These advancements are particularly important for quality control and standardization processes in the herbal medicine industry. With accurate and detailed genetic information, TGS ensures that the correct plant species and varieties are used in herbal products, ultimately boosting product safety, efficacy, and consumer confidence.

The integration of pan-genomics into TGS frameworks offers substantial advancements in plant identification [73]. Comprehensive pan-genomes of medicinal plant species allow for the identification of core and accessory genomic elements, including rare alleles and structural variations, that influence specialized metabolite biosynthesis and species-specific traits [74,75]. For instance, a pan-genome analysis of mung bean (*Vigna radiata* L.) revealed SVs and presence/absence variations in genes related to the photoperiodic flowering pathway, highlighting their role in influencing adaptation traits such as early flowering under specific environmental conditions [76]. Although still in its early stages, pan-genomic analysis in medicinal plants shows great potential for generating novel molecular markers to enhance the precision of plant identification.

In conclusion, TGS technologies offer advanced capabilities for identifying and characterizing the genomes of medicinal plants. By overcoming the limitations of NGS, these platforms provide deeper insights into genome complexity, SVs, and subtle genetic differences—critical for understanding medicinal plant properties. The inclusion of pan-genomics enhances these capabilities, providing a stronger basis for understanding genetic diversity in medicinal plants. Ongoing improvements in long-read technologies are expected to enhance affordability and accessibility, making TGS an increasingly vital tool for medicinal plant research, conservation, and quality control.

## 7. Integration of Multi-Omics Approaches for Comprehensive Identification

**Figure 3:**
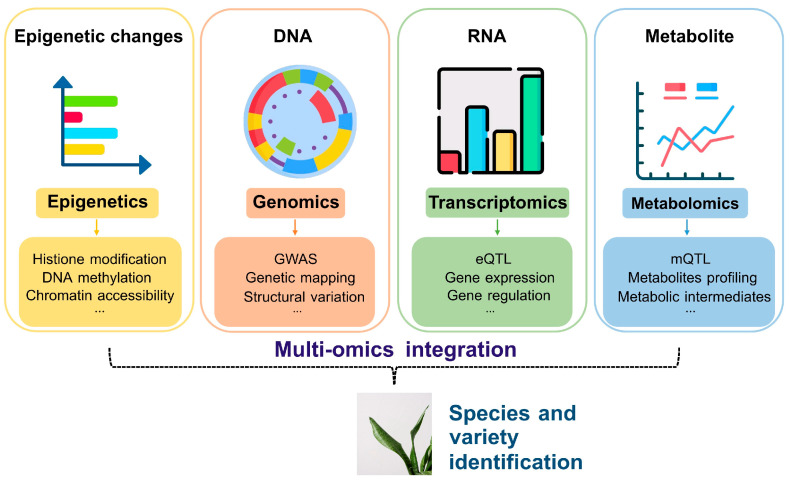
Integration of multi-omics approaches for comprehensive identification of medicinal plants.

Achieving the precise identification of medicinal plants requires integrating genomics with transcriptomics, metabolomics, and epigenetics [77]. Multi-omics approaches provide a holistic understanding of plant identity and function by combining various layers of biological information (Figure 3).

### 7.1. Combining Genomics, Transcriptomics, and Metabolomics

Integrating genomics, transcriptomics, and metabolomics greatly improves the precision of identifying medicinal plant species and varieties. Genomics offers insights into DNA sequences, while transcriptomics reveals gene expression dynamics, and metabolomics uncovers the biochemical compounds responsible for therapeutic effects [78]. By correlating these data, it is possible to distinguish between genetically similar plant varieties with distinct phenotypic or biochemical properties. For instance, transcriptome profiling can show how environmental factors affect the expression of genes involved in secondary metabolite production. In the case of *Artemisia annua* L. exposed to UV-B radiation, transcriptomic analysis revealed how transcription factors regulate artemisinin and flavonoid accumulation, offering insights into the complex regulatory networks underlying secondary metabolite biosynthesis [79]. Metabolomics further links these variations to differences in medicinal efficacy, enhancing the accuracy of medicinal plant identification [80].

### 7.2. The Role of Epigenetics and Gene Expression Profiling

Epigenetic modifications, such as DNA methylation and histone modifications, significantly affect plant phenotypic diversity without altering the underlying DNA sequence [81]. These modifications are crucial in the production of key secondary metabolites [65], providing an additional layer of information for distinguishing genetically similar varieties. By integrating epigenetic analysis with gene expression profiling, it is possible to differentiate varieties with identical genomic sequences but varying medicinal properties. This is particularly vital for ensuring consistent therapeutic outcomes, as subtle variations in epigenetic regulation can alter bioactive compound levels. For example, the identification of cytosine-5 DNA methyltransferase genes in *S. miltiorrhiza* Bunge revealed that DNA methylation regulates the biosynthesis of bioactive compounds, such as tanshinones and phenolic acids, through tissue-specific gene expression [82]. This highlights the importance of epigenetic mechanisms in maintaining the consistency of medicinal properties.

### 7.3. Integrating Sequencing Data with Chemical Profiling

Combining sequencing data with chemical profiling provides a robust method for authenticating medicinal plant species and ensuring product quality. Techniques such as HPLC, GC-MS, and LC-MS are used to identify bioactive compounds in plant extracts, and linking these chemical profiles with genomic and transcriptomic data helps verify plant authenticity and detect potential adulteration. For example, in *Saposhnikovia divaricata* (Turcz.) Schischk., correlating LC-MS-derived chemical profiles (such as flavonoid and coumarin content) with transcriptomic data provided key insights into how gene expression influences metabolite levels, ensuring the authenticity and consistent medicinal quality of cultivated versus wild varieties [83]. This approach is particularly useful for closely related species like *P. ginseng* C.A.Mey. and *Panax quinquefolius* L., where chemical composition is crucial for distinguishing varieties with similar morphological traits but differing pharmacological properties.

### 7.4. Integrating Genome Editing-Based Approaches

Advances in genome editing technologies have significantly enriched the toolkit for medicinal plant identification. The whole-genome analysis and genome editing (GAGE) method exemplifies an innovative approach by combining whole-genome data analysis with CRISPR/Cas12a-based genome editing [84]. GAGE involves two key steps: (1) analyzing the genome to identify species-specific target sequences and (2) using the CRISPR/Cas12a-based system to detect these sequences via fluorescence signals from the cleavage of a synthetic DNA reporter. This streamlined approach ensures precise and rapid species identification. GAGE has been successfully applied to differentiate *Crocus sativus* L. (saffron) from its adulterants and validated across diverse plant lineages, including angiosperms, gymnosperms, ferns, and lycophytes. Looking ahead, genome editing technologies in medicinal plant identification are expected to advance further with innovations such as multi-target detection for enhanced resolution, portable field-based diagnostic systems, integration with multi-omics data for comprehensive analysis, and applications to environmental DNA for biodiversity monitoring.

### 7.5. Integrating Other Innovative Approaches

Beyond the multi-omics approaches outlined above, several emerging technologies offer the potential to enhance the precision of plant identification. High-throughput chromosome conformation capture (Hi-C) technology enables the analysis of chromatin 3D structure, offering insights into the spatial architecture of genomes and its functional implications, which is crucial for understanding genome organization and its role in plant traits [85]. For instance, Hi-C technology provides a detailed map of the chromatin interactions in both wild-type and dwarf mutant peanut lines, revealing how changes in chromatin architecture may influence gene expression and contribute to the genetic mechanisms underlying dwarfism [86]. The combination of single-cell sequencing and ATAC-seq enables the investigation of gene expression dynamics and chromatin accessibility at the single-cell level [87]. This approach sheds light on cellular heterogeneity and responses to environmental stresses, providing a more refined method for identifying plant varieties and their adaptive potential. Recent studies using single-cell RNA-seq and ATAC-seq in *Arabidopsis* [88], rice [89], and peanut [90] have explored the significant impact of chromatin accessibility on gene expression and cellular heterogeneity, providing valuable models for studying the development and adaptation of medicinal plants. Moreover, the emergence of precision genome editing tools such as prime editing and base editing enables highly accurate modifications of plant genomes [91], reducing off-target effects and providing powerful tools for functional validation of plant traits linked to identification. For instance, the CRISPR/Cas9-mediated editing of uORFs in the *LsGGP2* gene in lettuce has led to enhanced oxidative stress tolerance and increased ascorbate content, highlighting the potential of genome editing in improving both nutritional and stress-related traits in medicinal plants [92,93]. Additionally, epigenome editing, by modifying DNA methylation and other epigenetic markers, offers a new layer of precision in regulating plant traits linked to environmental adaptability [94]. For instance, a recent study uses a dCas9-derived tool to accurately remove the H3K27me3 chromatin mark from a crucial developmental gene, illustrating how epigenome editing can directly modify gene expression and developmental pathways [95]. By integrating these technologies with other multi-omics data, a more comprehensive and accurate approach to medicinal plant identification can be achieved, enhancing efficiency, precision, and accessibility.

### 7.6. Challenges and the Need for Standardization

Despite their potential, multi-omics approaches face challenges related to standardization and cost. The lack of standardized protocols for data integration and interpretation often results in discrepancies between laboratories. Moreover, the absence of comprehensive multi-omics databases limits the ability to effectively cross-reference and interpret data. To overcome these barriers, developing advanced bioinformatics tools and standardized practices is essential to fully harness the benefits of multi-omics integration in medicinal plant identification.

In summary, integrating genomics, transcriptomics, metabolomics, and epigenetics provides a robust framework for accurate medicinal plant identification. Combining these approaches allows for capturing the complexity of plant identity and function, ensuring the quality and efficacy of herbal products. However, further efforts are needed to establish standardized protocols and develop comprehensive databases to make multi-omics approaches more accessible and effective for routine use.

## 8. Challenges, Future Directions, and Conclusions

Despite the significant advancements, several challenges still hinder the widespread adoption of sequencing technologies for medicinal plant identification. A major obstacle is the cost of high-throughput sequencing and the computational infrastructure needed for analysis. While sequencing costs have decreased substantially over time, the initial investment required to establish a fully functional sequencing and bioinformatics pipeline remains prohibitive for many research institutions, particularly in developing regions where medicinal plants are widely used. This financial hurdle is further exacerbated by the shortage of specialized bioinformatics expertise in many laboratories, making accurate data analysis and interpretation difficult.

Another critical challenge is the lack of standardized protocols and regulatory frameworks. Regulatory bodies in the herbal medicine industry have not yet fully integrated sequencing technologies into quality control, creating uncertainties about their acceptance. The lack of standardized procedures discourages industry investment, as the methods lack formal recognition. Moreover, inconsistencies in sample preparation, sequencing techniques, and data analysis pipelines contribute to variations across laboratories, further compromising the reliability and reproducibility of results.

In the future, several emerging technologies and strategies could help address these challenges and transform medicinal plant identification. AI-assisted bioinformatics holds great potential for automating the analysis of large sequencing datasets, reducing the need for specialized expertise in data interpretation. By employing machine learning algorithms, AI can efficiently identify patterns in sequencing data, facilitating rapid and accurate species identification. Additionally, the development of portable sequencing devices, such as the Oxford Nanopore MinION, offers opportunities for real-time, on-site plant identification, which is particularly valuable in remote areas. These portable technologies could revolutionize quality control and field research by enabling rapid identification without requiring extensive laboratory infrastructure.

Another promising avenue is the development of integrated pipelines that combine sequencing, data analysis, and reporting into a streamlined workflow, making these technologies more accessible for routine use in the herbal industry. Cloud computing and data-sharing platforms could further enhance sequencing capabilities by providing smaller laboratories with the computational resources necessary to analyze complex datasets. Establishing shared, comprehensive reference databases is also crucial for enhancing the reliability of molecular identification. Standardized databases that integrate genomic, transcriptomic, and metabolomic data would significantly improve the accuracy of plant identification and facilitate the widespread adoption of sequencing technologies.

In conclusion, modern sequencing technologies have the potential to revolutionize medicinal plant identification by providing more accurate, scalable, and reliable methods than traditional approaches. To fully integrate these technologies into quality control and conservation practices, challenges such as cost, regulatory acceptance, and data complexity must be addressed. Advancements in integrated platforms, optimized bioinformatics tools, and standardized protocols will be essential in advancing this field. The future of medicinal plant identification lies in the seamless integration of these cutting-edge technologies with traditional practices, ensuring the sustainable use and conservation of valuable medicinal plant resources.

## Figures and Tables

**Table 1:** Comparison of molecular markers, DNA barcoding, next-generation sequencing, and third-generation sequencing for plant identification.

Feature/Method	Molecular Markers	DNA Barcoding	Next-Generation Sequencing	Third-Generation Sequencing
**Technique**	SSR, RAPD, AFLP, SNP	*rbcL*, *matK*, ITS	Illumina, Ion Torrent	PacBio, Oxford Nanopore
**Data scope**	Specific loci	Specific gene regions	Large genomic regions, whole-genome	Ultra-long reads, whole-genome
**Advantages**	Cost-effective, simple	Rapid, consistent results	Scalability, high accuracy	Long-read data, structural analysis
**Limitations**	Reproducibility issues, labor-intensive	Limited to species level	High cost, computational demands	Higher cost, complex data handling
**Scalability**	Moderate	High	Very high	Moderate
**Bioinformatics requirement**	Low	Low	High	Very high
**Suitability for mixed samples**	Limited	Limited	Excellent (metabarcoding)	Moderate
**Discriminatory power**	Moderate (depends on marker)	Moderate (species level)	High (genome-wide)	Very high (structural variation)

## Data Availability

Not applicable.
